# Identification of synthetic lethality of PRKDC in MYC-dependent human cancers by pooled shRNA screening

**DOI:** 10.1186/1471-2407-14-944

**Published:** 2014-12-13

**Authors:** Zongxiang Zhou, Manishha Patel, Nicholas Ng, Mindy H Hsieh, Anthony P Orth, John R Walker, Serge Batalov, Jennifer L Harris, Jun Liu

**Affiliations:** Genomics Institute of the Novartis Research Foundation, 10675 John Jay Hopkins Drive, San Diego, CA 92121 USA

**Keywords:** PRKDC, MYC, Synthetic lethality, RNAi screen, Cancer, DNA damage, DNA repair

## Abstract

**Background:**

MYC family members are among the most frequently deregulated oncogenes in human cancers, yet direct therapeutic targeting of MYC in cancer has been challenging thus far. Synthetic lethality provides an opportunity for therapeutic intervention of MYC-driven cancers.

**Methods:**

A pooled kinase shRNA library screen was performed and next-generation deep sequencing efforts identified that PRKDC was synthetically lethal in cells overexpressing MYC. Genes and proteins of interest were knocked down or inhibited using RNAi technology and small molecule inhibitors, respectively. Quantitative RT-PCR using TaqMan probes examined mRNA expression levels and cell viability was assessed using CellTiter-Glo (Promega). Western blotting was performed to monitor different protein levels in the presence or absence of RNAi or compound treatment. Statistical significance of differences among data sets were determined using unpaired *t* test (Mann–Whitney test) or ANOVA.

**Results:**

Inhibition of PRKDC using RNAi (RNA interference) or small molecular inhibitors preferentially killed MYC-overexpressing human lung fibroblasts. Moreover, inducible PRKDC knockdown decreased cell viability selectively in high MYC-expressing human small cell lung cancer cell lines. At the molecular level, we found that inhibition of PRKDC downregulated MYC mRNA and protein expression in multiple cancer cell lines. In addition, we confirmed that overexpression of MYC family proteins induced DNA double-strand breaks; our results also revealed that PRKDC inhibition in these cells led to an increase in DNA damage levels.

**Conclusions:**

Our data suggest that the synthetic lethality between PRKDC and MYC may in part be due to PRKDC dependent modulation of MYC expression, as well as MYC-induced DNA damage where PRKDC plays a key role in DNA damage repair.

**Electronic supplementary material:**

The online version of this article (doi:10.1186/1471-2407-14-944) contains supplementary material, which is available to authorized users.

## Background

Targeted therapies inhibiting druggable gain-of-function oncogenes such as BCR-ABL, EGFR, HER2, ALK, and mutant BRAF have shown striking benefits in cancer patients in the clinic. However, many challenges remain for targets which lack druggable domains, such as RAS and MYC, two of the most frequently deregulated human oncogenes [1–4].

MYC family members (c-, L-, and N-) are ubiquitously expressed in mammals, exhibit similar biological functions, and are under tight regulation throughout development and adulthood. MYC is found downstream of a number of growth factor receptors, acting as a central hub directing signals that favor various aspects of cell growth, such as cell proliferation and resistance to apoptosis [5]. Rather than a direct mutation of MYC, overexpression of this oncoprotein is the major underlying mechanism of action for its tumor-promoting properties [5].

Due to the lack of a druggable domain, it has been challenging to develop small molecule inhibitors that target MYC itself, or disrupt MYC-mediated protein-protein or protein-DNA interactions [6, 7]. Additionally, MYC expression in normal cells including regenerative tissues such as the gastrointestinal tract, skin and bone marrow, raises concern for achieving an acceptable therapeutic index in MYC-targeted therapies. To this end, a dominant-negative MYC mutant (OmoMyc) was developed to inhibit MYC’s interaction with its key binding partner, MAX [8–10]. The disruption of this heterodimerization resulted in inhibition of MYC-dependent target gene expression [11–13]. OmoMyc-mediated MYC inhibition led to a dramatic decrease in tumor-burden in a murine Kras lung cancer model, with only mild side effects [14, 15], suggesting differential MYC dependency between tumor and normal tissues.

For non-druggable targets like MYC, synthetic lethality offers a unique opportunity for therapeutic intervention. The principle of synthetic lethality is that mutation of either gene ‘A’ or ‘B’ alone is non-detrimental to the cell, while mutation of both genes leads to cell death [16, 17]. For example, the Parp1 inhibitor is synthetic lethal to cells harboring *BRCA1/2* mutations [18–20]. In phase II clinical trials, the PARP1 inhibitor, Olaparib, showed a 41% response rate as a single agent in breast and ovarian cancer patients with *BRCA1*/*BRCA2* mutations [21, 22]. Towards identifying novel synthetic lethality targets, RNA interference (RNAi) technology has made it feasible to investigate a large cohort of genes for loss-of-function effects [23].

In our quest to reveal novel synthetic lethal genes in the context of MYC-deregulated cancers, we conducted a pooled shRNA screen using isogenic cell lines. We identified and confirmed PRKDC (Protein Kinase, DNA-activated, Catalytic polypeptide), a protein kinase with a major role in non-homologous end joining (NHEJ) DNA repair [24, 25]), as a novel synthetic lethal target in MYC-overexpressing lung cancer cells. We found that downregulation of PRKDC expression in MYC-overexpressing cells led to a significant reduction of MYC-dependent cell proliferation. Additionally, PRKDC can modulate MYC mRNA and protein expression levels. Moreover, our data reconfirmed that overexpression of MYC family proteins induced DNA double-strand breaks, and we further demonstrated an increase in DNA damage upon PRKDC inhibition in cells overexpressing MYC. Altogether, our results indicate that PRKDC may be critical in MYC-driven oncogenesis, and support PRKDC as a potential synthetic lethal target for MYC.

## Methods

### Antibodies and western blot analyses

The following primary antibodies were used in western blot: γH2AX (1:2000 dilution, Millipore, cat# 05–636), GAPDH (1:1000 dilution, Cell signaling, cat# 3683), gamma-tubulin (1:5000, Thermo Scientific cat#MA1-850), c-MYC (1:1000, Cell signaling, cat# 5605), N-MYC (1:1000, Cell signaling, cat# 9405), L-MYC (1:1000, R&D systems, cat# AF4050) and PRKDC (1:200 dilution, Santa Cruz, cat# sc-9501). For immunoblots, cells were lyzed with either CelLytic M cell lysis buffer (Sigma) or RIPA buffer (Sigma cat#89900) and equal amounts of protein lysates were mixed with XT sample buffer and reducing agent (Bio-Rad), separated by SDS Criterion precast gels (Bio-Rad), and transferred to a PVDF membrane (Bio-Rad). Proteins were detected with primary antibodies and horseradish peroxidase-conjugated secondary antibodies by using SuperSignal West Dura Extended Duration Substrate (Thermo Scientific).

### Plasmids and chemical inhibitors

shRNAs pLKO.1 lentiviral plasmids used for non-targeting shRNA control (SHC002) and human PRKDC knockdown were purchased from Sigma. The shRNA clones used for targeting PRKDC were TRCN0000197152 (shPRKDC#1), TRNC0000196328 (shPRKDC#2), TRCN0000195491 (shPRKDC#3), TRCN0000194985 (shPRKDC#4) and TRCN0000194719 (shPRKDC#5). Non-targeting control shRNA and three PRKDC shRNAs (shPRKDC#1, shPRKDC#3 and shPRKDC#5) were cloned into the inducible pLKO-Tet-On puromycin vector as previously described [26]. pCDH-CMV-MCS-EF1-Puro lentivector was purchased from System Bioscience. pCDH-c-MYC, pCDH-L-MYC1, pCDH-L-MYC2 and pCDH-N-MYC vectors were generated by cloning protein coding sequences of human c-MYC, L-MYC isoform1, L-MYC isoform2 and N-MYC into pCDH-CMV-MCS-EF1-Puro lentivector. Etoposide, NU-7441, and KU0060648 were obtained from Sigma (cat# E1383), Tocris Cookson Inc. (cat# 3712), and Axon Medchem BV (cat# Axon 1584), respectively. The proteasome inhibitor, MG132, was obtained from Sigma (cat#C2211).

### Cell culture

All cell lines were cultured in a humidified incubator at 37°C with 5% CO_2_. The following cell lines were obtained from ATCC: HEK 293 T, WI-38, WI-38 VA 13, Daudi, EB1, HS604T, HS616T, HuT 102, MC116, Namalwa, H1963, H196, H209, H526, H524, H82, H69, Raji, SW1271, and TO175T. DEL, L428, SU-DHL-10, WSU-DLCL2, Jurkat, DND41 and SR768 were purchased from DSMZ. A4/Fuk was obtained from JCRB Cell Line Bank. OCI-Ly3 was a kind gift from Dr. Mark Minden (University Health Network Toronto). The human lung fibroblast cell lines WI-38 and WI-38 VA13 were routinely cultured in EMEM supplemented with 10% Fetal Bovine Serum (FBS). SCLC cell lines H1963, H196, SW1271, H209, H526, H524, H82 and H69 were maintained in RPMI 1640 with 10% FBS. The lymphoma cell lines EB1, Daudi, Raji, A4/Fukuda, Jurkat, DND41, WSU-DLCL2, DEL, HUT102, Namalwa and L428 were cultured in RPMI 1640 with 10% FBS. MC116 and SU-DHL-10 cells were maintained in RPMI 1640 with 20% FBS. OCI-Ly3 cells were cultured in IMDM with 20% FBS. The SR786 cell line was cultured in RPMI 1640 with 15% FBS. HS604T cells were maintained in DMEM with 10%FBS and 2 mM Glutamine. HS616T and TO175T cell lines were cultured in DMEM with 10% FBS. HEK 293 T cells were maintained in DMEM with 10% FBS. All cell lines were maintained with a cocktail of penicillin and streptomycin (Gibco).

### Lentivirus and infection

Lentivirus packaging and infection was performed according to the established protocols from the RNAi consortium (http://www.broadinstitute.org/rnai/public/resources/protocols). Briefly, shRNA-encoding plasmids were transfected into 293 T cells with packaging plasmids encoding gag-pol-rev and vesicular stomatitis virus envelope glycoprotein using Fugene6 (Roche). Growth media was changed the following day and lentivirus-containing supernatants were harvested 2–3 days after transfection, filtered and used to infect cells in the presence of 8 μg/ml polybrene (Sigma).

### Generation of stable inducible shRNA-expressing cell lines

To generate tet-inducible stable cell lines, SCLC cell lines were transduced with lentivirus expressing tet-inducible shRNA against PRKDC or non-targeting control in the presence of 8 μg/ml polybrene (Sigma). Medium was changed the following day and cells were selected by puromycin and expanded for at least one week before performing experiments. Induction of shRNA expression was performed by addition of 100 ng/ml doxycycline (Clontech) to the cell culture medium.

### Cell viability assay

Cell viability assay was performed using CellTiter-Glo (Promega) according to manufacturer’s instructions. Briefly, cells were seeded at 3000 cells/well in a 96-well plate (6 wells/sample) in the presence and absence of doxycycline. CellTiter-Glo measurements were taken at several time points to track cell proliferation.

### RNA extraction and quantitative RT-PCR (TaqMan)

Total RNA was isolated using the Qiagen RNeasy kit according to the manufacturer’s instructions. cDNA was generated from 0.5 μg total RNA using High Capacity cDNA Reverse Transcription kit (ABI). TaqMan probes include c-MYC (Hs00153408_m1), N-MYC (Hs00232074_m1), L-MYC1 (Hs00420495_m1), L-MYC2 (Hs01921478_s1) and PRKDC (Hs00179161_m1) (ABI). TaqMan PCR was performed by using the ABI PRISM 7900 HT Sequence Detection System. All experiments were performed in triplicate and normalized to GAPDH.

### Pooled shRNA screening

pGW-LentLox3.7 lentiviral-based human kinome shRNA library containing 1300 shRNAs (targeting 500 human kinase genes, 2–3 shRNAs per gene) was designed and constructed by Genomics Institute of the Novartis Research Foundation (GNF). 1300 lentiviral kinase shRNA plasmids and non-targeting control shRNA plasmids were combined at equal concentration in one pool and used to generate pooled lentivirus. For screening, three WI-38 isogenic cell lines (overexpressing L-MYC1, L-MYC2 or empty vector control) were infected with the pooled lentivirus using a MOI of 0.5. Medium was changed the following day and twenty-four hours later, 2×10^6^ cells were harvested as day 1 sample. 2×10^6^ cells were further cultured for 14 days (day 14 sample). Genomic DNA from day 1 and day 14 samples was isolated by using a DNeasy blood & tissue kit (Qiagen). Deep-sequencing template libraries were generated by PCR amplification of shRNA hairpin from genomic DNA. PCR products were purified by using a QIAquick PCR purification kit (Qiagen). After purification, PCR products from each sample were quantified, pooled at equal proportions and analyzed by high-throughput sequencing (Eureka).

### Statistical analysis

All numerical data are shown as mean ± SD or SEM. Error bars on all graphs represent the standard deviation or SEM between measurements. Statistical significance of differences among data sets were determined using unpaired *t* test (Mann–Whitney test) or one-way ANOVA using PRISM 6 (GraphPad, San Diego, CA). *P* values are indicated with asterisks (****P ≤0.0001; ***P ≤ 0.001; **P ≤ 0.01; *P ≤ 0.05).

## Results

### Large scale RNAi screen using L-MYC-overexpressing cells uncovers PRKDC as a novel candidate for synthetic lethality

In our pursuit to uncover novel synthetic lethality targets of the MYC signaling pathway, we conducted a large scale loss-of-function RNAi screen in L-MYC-overexpressing lung fibroblasts and their isogenic controls. Here, we used an in-house pooled kinase shRNA library (1279 shRNA constructs targeting 486 human kinase genes). We screened the lung fibroblast cell line, WI-38, stably expressing empty vector, L-MYC isoform 1 (L-MYC1) or L-MYC isoform 2 (L-MYC2) (overexpressing an inactive L-MYC transcriptional variant), with our lentiviral shRNA library. Genomic DNA was isolated from each sample and subjected to next generation sequencing. The change in relative abundance of each shRNA among the three WI-38 cell lines was analyzed.

**Figure 1:**
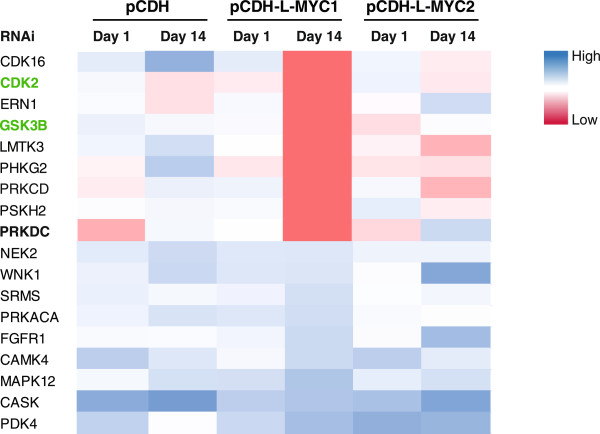
**Large-scale RNAi screen identifies PRKDC as a MYC synthetic lethal gene.** Heat map of relative counts of 18 shRNAs for WI-38 cell lines stably-expressing empty vector (pCDH), L-MYC1 or L-MYC2, after infection with a pooled kinase shRNA library. Blue = high expression, red = low expression; intensity of color represents relative counts per million total reads. Highlighted in green (CDK2 and GSK3B) are previously published synthetic lethal partners of MYC.

Our primary screening hits include both novel candidates, and previously reported genes functioning as synthetic lethal partners with MYC, such as CDK2 and GSK3B; CDK2 and GSK3B served as positive controls in this screen [27, 28] (Figure 1 and Additional file 1: Table S1). We identified a small cohort of potential MYC synthetic lethal partners as highlighted in Figure 1. Among the novel candidates, we were specially intrigued by the reduction in PRKDC shRNA levels in L-MYC1-overexpressing cells as compared to the isogenic vector control and L-MYC2-overexpressing cell lines (Figure 1 and Additional file 1: Table S1). PRKDC encodes for the catalytic subunit of the DNA-dependent serine/threonine-protein kinase (also widely known as DNA-PKcs). It functions as a molecular sensor for damaged DNA and engages in DNA non-homologous end joining (NHEJ) required for double-strand break (DSB) repair and somatic recombination [29–31]. Previous research reported that downregulation of PRKDC by siRNA leads to a decrease of MYC protein level in Hela cells [32]. Moreover, MYC-induces upregulation of γH2AX, a protein heavily implicated in DSB [33, 34]. We hypothesized that since MYC-driven cancer cells may be more dependent on DNA damage pathways, PRKDC is an attractive candidate for a druggable synthetic lethal gene.

### PRKDC knockdown in L-MYC overexpressing lung fibroblasts and cancer cells decreases cell viability

**Figure 2:**
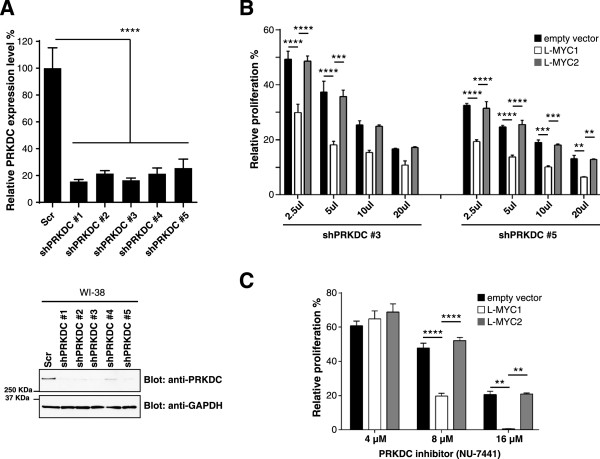
**PRKDC gene suppression in MYC-overexpressing human lung fibroblast cells decreases cell viability. A)** Gene and protein knockdown efficiency with independent shRNA clones against PRKDC quantified by RT-PCR and immunoblotting, respectively, in WI-38 cells. Protein was analyzed via immunoblotting for PRKDC (anti-PRKDC) and GAPDH (anti-GAPDH). **B)** Stable WI-38 cell lines were exposed to increasing amounts of lentivirus expressing PRKDC shRNAs and subjected to a cell viability assay after 6 days. **C)** Stable WI-38 cell lines were treated with varying concentrations of a PRKDC inhibitor, NU-7441, for 3 days and subjected to a cell viability assay. Data are shown as mean ± SD. Statistical analysis using one-way ANOVA; ****P ≤0.0001; ***P ≤ 0.001; **P ≤ 0.01.

Next, we wanted to reconfirm the functional consequence of PRKDC suppression in the context of L-MYC overexpression. The gene knockdown efficiency of five shRNAs against PRKDC was confirmed by quantitative RT-PCR and Western blot analysis in WI38 cells (Figure 2A). Two shRNAs (shPRKDC-3 and shPRKDC-5) demonstrating good reduction of PRKDC expression levels were chosen for functional experiments. Titration experiments using PRKDC shRNA-expressing lentiviruses demonstrated a specific decrease in cell viability in L-MYC1-overexpressing WI-38 cells as compared to the control cells (Figure 2B). Significantly, our results showed PRKDC dependency in cells expressing active L-MYC, but not in cells expressing an inactive L-MYC isoform. Similar and reproducible results were obtained using two independent shRNAs (shPRKDC-3 and shPRKDC-5) (Figure 2B). Moreover, we subjected WI-38 cells to PRKDC inhibition using a potent and selective PRKDC inhibitor, NU-7441 [35]. With increasing PRKDC inhibitor concentrations, we noted a marked decrease in cell viability for L-MYC1-overexpressing cells when compared to controls (Figure 2C). All cell lines displayed similar growth curves in the absence of the PRKDC inhibitor (Additional file 2: Figure S1). Our data suggests that the MYC oncogene confers a dependency on PRKDC for cell viability and makes cells sensitive to PRKDC inhibition.

### Inducible PRKDC knockdown decreases human SCLC cell proliferation and is dependent on high *MYC*expression levels

**Figure 3:**
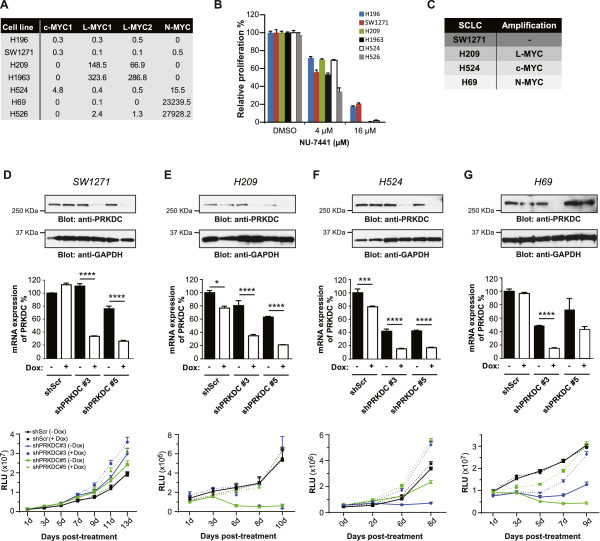
**Inducible PRKDC knockdown decreases human SCLC cell proliferation and is dependent on high** ***MYC*** **expression levels. A)** *MYC* family gene expression levels in non-isogenic SCLC cell lines (as compared to *MYC* family gene expression levels in 293 T cells). **B)** SCLC cell lines with different levels of *MYC* gene expression were treated with varying concentrations of a PRKDC inhibitor, NU-7441, for 3 days and subject to a cell viability assay. **C)** *MYC* gene amplification status in different SCLC cell lines. **D**-**G)** SCLC cell lines were subjected to inducible PRKDC downregulation with three independent shRNA clones and knockdown was confirmed via immunoblotting and RT-PCR after doxycycline exposure. Protein was analyzed via immunoblotting for PRKDC (anti-PRKDC) and GAPDH (anti-GAPDH). The SCLC cell lines were exposed to doxycycline for 6–13 days and then subjected to a cell viability assay. Data are shown as mean ± SD. Statistical analysis using one-way ANOVA; ****P ≤0.0001; ***P ≤ 0.001; **P ≤ 0.01; *P ≤ 0.05.

To further test our findings in cancer cells, we investigated PRKDC suppression in a panel of human small-cell lung cancer (SCLC) cell lines with differential levels of *MYC* family gene amplification and mRNA expression. It was reported that the *L-MYC* gene was amplified in H209 and H1963 cell lines, *c-MYC* was amplified in H524 and *N-MYC* was amplified in the H526 cell line. None of the *MYC* family genes were amplified in H196 and SW1271. By TaqMan RT-PCR, we confirmed that mRNA expression levels of *MYC* family genes (as compared to 293 T cells and this panel of SCLC lines) are correlated with their gene amplification status (Figure 3A). Functionally, inhibition of PRKDC using NU-7441 preferentially killed cells with either *L-MYC* (H209 and H1963), *c-MYC* (H524) or *N-MYC* (H526) overexpression, compared to cell lines without *MYC* family gene overexpression (H196 and SW1271) (Figure 3B).

To confirm inhibition of PRKDC preferentially kills SCLC tumor cells with *MYC* overexpression, we used shRNAs to knockdown PRKDC. Since many SCLC cell lines grow in suspension and form big clumps, evaluation of PRKDC knockdown effects on cell growth using constitutive shRNAs was difficult. We therefore decided to use doxycycline-regulated inducible shRNAs for PRKDC downregulation for these experiments. Briefly, cancer cells were stably infected with inducible TetOn lentiviruses where the expression of PRKDC shRNA was under the control of the doxycycline promoter. *In vitro*, PRKDC knockdown was confirmed via PRKDC-specific TaqMan and Western blot analysis in each SCLC cell line tested in response to doxycycline treatment (Figure 3D-G). Different SCLC cell lines with differential *MYC* expression patterns (Figure 3C) were exposed to doxycycline for 7–13 days *in vitro* (Figure 3D-G). While inducible knockdown of PRKDC in SW1271 (cell line with no *MYC* gene amplification) had minimal effect on growth inhibition (Figure 3D), the same treatment of H209 cells (*L-MYC* gene amplification) greatly inhibited cell proliferation (Figure 3E). Similar results of cell proliferation inhibition were observed in H524 (*c-MYC* gene amplification) and H69 (*N-MYC* gene amplification) cell lines upon PRKDC knockdown (Figure 3F-G). The data is consistent with our hypothesis that PRKDC loss-of-function synthetic lethality is reliant on high *MYC* expression levels.

In order to further validate PRKDC dependency in other MYC-driven cancers, we chose to examine c-MYC in human lymphoma cell lines using the potent PRKDC inhibitors, NU-7441 and KU0060648 [36]. We selected a panel of human lymphoma cell lines and divided them into *c-MYC* high and low groups (BioGPS database). The IC_50_ of the two PRKDC inhibitors were measured in a cell proliferation assay in these cell lines (Additional file 3: Figure S2A). Cell lines with high *c-MYC* gene expression levels were more responsive to NU-7441 as compared to the low *c-MYC* expression cohort, displaying IC_50_ values of 2.09 ± 0.56 μM and 13.76 ± 5.18 μM, respectively (Additional file 3: Figure S2B). A similar IC_50_ trend for KU0060648 was observed in these cells (1.09 ± 0.23 μM and 9.27 ± 4.71 μM, respectively) (Additional file 3: Figure S2B). Together, our results suggest that the MYC oncogenic effect is dependent on PRKDC expression in multiple human cancers with high *MYC* expression levels.

### Suppression of PRKDC gene expression decreases c-MYC protein abundance in cancer cell lines

Our data suggested synthetic lethality of PRKDC in MYC-overexpressing cancers. We next wanted to gain insight into the underlying mechanism. It was reported that PRKDC can induce phosphorylation of MYC at various serine residues and modulate its stability [37]. Moreover, it has been observed that knockdown of PRKDC via RNAi reduces MYC protein stability in Hela cells [32, 38].

**Figure 4:**
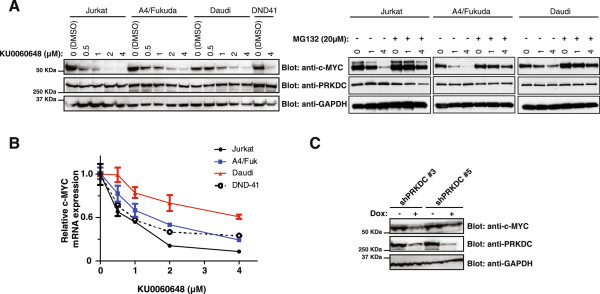
**MYC mRNA and protein levels are negatively affected by PRKDC gene suppression in cancer cell lines. A)** Different lymphoma cell lines were treated with increasing concentrations of the PRKDC inhibitor, KU0060648, and a proteasome inhibitor, MG132. After a 4 h drug exposure time, c-MYC protein levels were analyzed via immunoblotting with an anti-MYC antibody. PRKDC and GADPH protein levels were also monitored with anti-PRKDC and anti-GADPH antibodies, respectively. **B)** Cells from A were analyzed by RT-PCR for relative c-MYC mRNA expression levels. **C)** The H82 cell line expressing inducible PRKDC shRNA was exposed to doxycycline for 3 days. c-MYC, PRKDC and GADPH protein levels were analyzed by immunoblotting with anti-MYC antibody, anti-PRKDC and anti-GADPH antibodies, respectively. Gene knockdown efficiency with independent shRNA clones against PRKDC was quantified by RT-PCR as was relative c-MYC mRNA expression levels in these cell lines.

We examined c-MYC protein expression levels in lymphoma cell lines treated with the PRKDC inhibitor, KU0060648. After a 4 h drug treatment, we noted a significant and dose-dependent decrease in c-MYC protein levels in multiple lymphoma cell lines, correlating with a concurrent decrease in c-MYC mRNA expression (Figure 4A and B, respectively). Furthermore, using our inducible PRKDC shRNA knockdown system in H82 SCLC cells, we observed a concomitant reduction in c-MYC protein when cells were exposed to doxycycline (Figure 4C). Interestingly, the proteasome inhibitor, MG132, did not completely rescue the effect of PRKDC inhibition on MYC abundance (Figure 4A). Similar results were observed with another PRKDC inhibitor, NU-7441, when used in conjunction with MG132 (Additional file 4: Figure S3). Collectively, these data indicate that PRKDC modulates c-MYC mRNA and protein expression in these cancer cell lines; the effect on MYC protein abundance is at least in part through reduction of MYC mRNA.

### Overexpression of MYC family proteins induces double-strand breaks in DNA in a SV40-transformed human lung cell line

**Figure 5:**
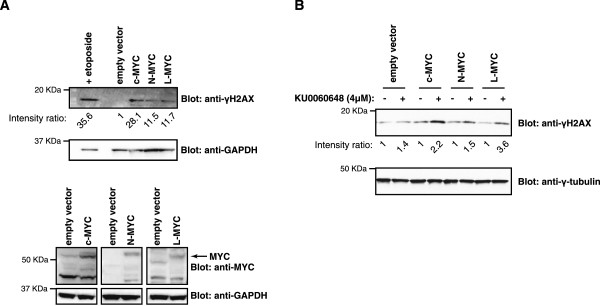
**MYC overexpression induces double-strand breaks in DNA. A)** A SV40-transformed human lung cell line, WI-38 VA13, stably-expressing c-MYC, L-MYC, or N-MYC, were lyzed and immunoblots were analyzed for phosphorylated histone H2AX (anti-γH2AX) and GADPH (anti-GADPH). As a positive control for DNA damage detection, parental cells were treated with etoposide and analyzed via immunoblotting. Cell lysates were also analyzed for overexpression of MYC variants. **B)** The same cells lines as in A were treated with the PRKDC inhibitor, KU0060648, for 8 h, lyzed and immunoblotted for phosphorylated histone H2AX (anti-γH2AX) and γ-tubulin (anti-γ-tubulin). Signal intensities of immunoblots were quantified using ImageJ.

In addition to the modulation of MYC protein and mRNA abundance by PRKDC, there is further interplay between the two proteins during DNA damage and repair processes. It was reported that overexpression of c-MYC induces DNA DSB [33, 39]. Our hypothesis is that in cancer cells, MYC overexpression induces DSBs and PRKDC plays a pivotal function in repairing this DNA damage, leading to cancer cell survival. Therefore, we overexpressed MYC in WI-38 VA13 cells, a SV40-transformed human lung cell line, and monitored for phosphorylated histone H2AX (γH2AX). γH2AX is a commonly used biomarker of DNA damage, which quickly accumulates in the cell following DSB induction to elicit amplification of the DNA damage response signaling cascade [40–42]. Our results showed that overexpression of MYC induced a substantial increase in γH2AX in WI-38 VA13 cells (Figure 5), which is consistent with previous reports [33, 39]. This was a universal finding for all MYC family members (c-, L-, and N-MYC) (Figure 5). As a positive control for DNA damage detection, we treated cells with Etoposide, a cytotoxic drug which causes DNA damage [43], and observed an increase in γH2AX expression (Figure 5). Additionally, inhibition of PRKDC in MYC-overexpressing cells further increased γH2AX levels. Our results suggest that, mechanistically, the synthetic lethality observed between PRKDC suppression and MYC overexpression may in part be due to the reliance of MYC-expressing cancer cells on PRKDC-mediated DNA damage repair.

## Discussion

The MYC family encodes transcription factors which play a critical role in diverse biological and pathophysiological processes. Amplification of the MYC oncogene commonly occurs in various types of human cancers. Not only has MYC been established as a ‘driver’ oncogene capable of initiating tumor formation, it has also been demonstrated that tumors become addicted to MYC and require MYC for tumor maintenance [44]. Multiple *in vivo* MYC-overexpression cancer murine models further support MYC as a therapeutic target [44]. Thus, due to MYC’s critical role in human cancer, mounting research efforts have been made to identify potential therapies for MYC-driven cancer. However, pharmacologic inhibition of MYC function has proven challenging, partially due to the absence of an apparent druggable domain in MYC transcription factors. Modulators that regulate MYC gene expression or protein abundance would be alternative therapeutic targets. Brd4, an epigenetic regulator of MYC, has been identified as a promising drug target for MYC-driven cancer [45]. Downregulation of MYC transcription by BET inhibitor, JQ1 compound, resulted in significant anti-tumor activity in mouse models [46, 47]. This is consistent with the notion that MYC overexpression in cells leads to oncogene addiction, a phenomenon where a tumor becomes reliant on a single dominant oncogene for growth and survival. Thus, MYC reduction through pharmacological intervention provides potential strategies to target MYC-driven cancer.

Studies to target MYC via synthetic lethality have reported multiple MYC synthetic lethal partners, such as ARK5, ATR, AURKB, CDK1/2, CHK1, CSNK1ϵ, DR5, GSK3B, and SAE1/2 [6, 27, 28, 48–54]. However, all these candidates remain to be validated in the clinic.

Pooled shRNA technology has made substantial progress in the last several years, with more sophisticated shRNA pools, increased deep sequencing capacity and reduced cost. Herein, we report identification of a synthetic lethality link between PRKDC and MYC through pooled shRNA screening and demonstrate inhibition of PRKDC preferentially kills MYC-overexpressing tumor cells.

PRKDC is a vital component of NHEJ DNA repair. Germ-line loss-of-function PRKDC mutations lead to disruption of T or B cell development and a severely compromised immunodeficiency phenotype in humans and mice [55, 56]. Nevertheless, PRKDC is not essential in model organisms, demonstrated in both genetically engineered and spontaneous animal models [55, 57–60], which may afford a potential safe therapeutic window for future drug development. To this end, in pre-clinical animal models, PRKDC inhibitors such as NU-7441 and KU0060648, demonstrated increased efficacy with a good with tolerability *in vivo* [36, 61]. Importantly, the dual PI3K/mTOR inhibitor, BEZ235, has displayed robust anti-PRKDC activities and inhibition of tumor growth in pre-clinical mouse models; CC-115 also has a similar mTOR/PRKDC dual inhibition activity [62–65]. Currently, clinical trials are underway with BEZ235 and CC-115 to determine its efficacy and safety in human patients [66]. It will be of great interest to dissect if its PRDKC activity could contribute to its tumor efficacy.

**Figure 6:**
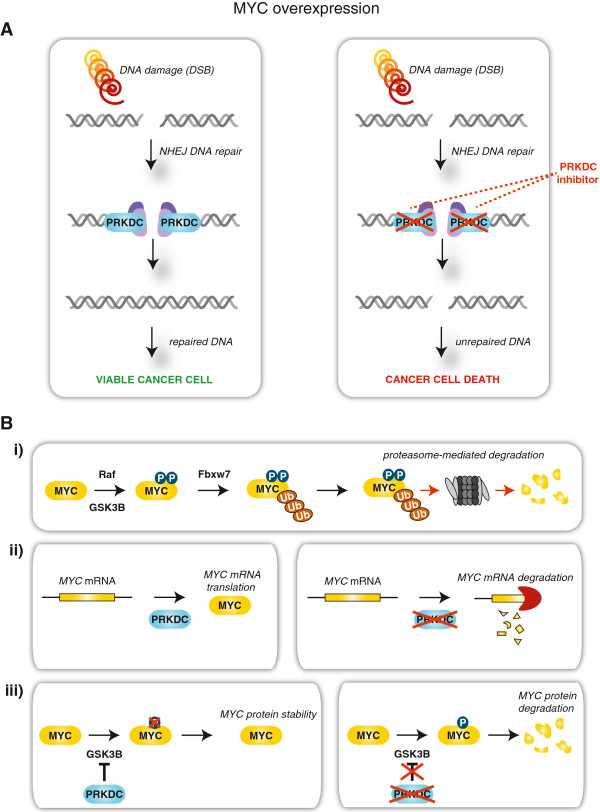
**Proposed model for synthetic lethality between MYC and PRKDC. A)** In MYC-driven cancer cells, the overexpression of MYC leads to DNA damage and creates a dependence on DNA repair machinery for cancer cell survival. Double strand breaks (DSBs) induce non-homologous end joining (NHEJ) DNA repair mechanisms to correct for DNA insult. PRKDC is a major player during NHEJ repair, and along with other key components, will restore the impaired DNA allowing for cancer cell viability. In these same cells, exposure to anti-PRKDC drug treatments would ultimately lead to PRKDC inhibition, compromised NHEJ repair and cell death. **B)** PRKDC has also been implicated in *MYC* gene regulation. i) In a normal setting, MYC protein is phosphorylated through RAF- and AKT-mediated signaling cascades, resulting in its FBW7-mediated ubiquitination, and subsequent proteasomal degradation. ii) In cancer cells, at the gene level, the inhibition of PRKDC protein decreases *MYC* expression, potentially through a direct effect or epigenetic mechanism(s). iii) Additionally in cancer cells, PRKDC can phosphorylate AKT, which results in the inhibition of GSK3β and subsequent MYC degradation. Therefore, interference with PRKDC function(s) decreases the stability of MYC protein. This form of genotypic cytotoxicity represents synthetic lethality that selectively targets cancer cells while leaving normal cells unscathed, and offers a potential for wider therapeutic windows for cancer therapies.

At the cellular level, MYC-induced γH2AX protein upregulation, a hallmark for DSB and replicative stress where PRKDC has an essential function, suggesting that MYC-driven cancer cells may be more dependent on DNA damage response components. MYC expression induces DSB formation, which if left unchecked, results in DNA damage and cell death [33, 34] (Figure 6). We hypothesize that MYC-overexpressing cancer cells may become more reliant on the DNA damage repair machinery of PRKDC for survival. Suppression of this DNA repair pathway would then lead to cancer cell death (Figure 6).

Consistent with our data, other components in the DNA damage response (DDR) pathway, such as ATR, are also implicated in MYC synthetic lethality [50]. In genetically engineered mouse models, reduction of ATR prevented MYC-driven lymphomas or pancreatic tumors [50]. In contrast, genetic ablation of ATR had no effect in Kras G12V-driven pancreatic tumor models [50]. Mechanistically, DSB initiates a signaling cascade through ATR and ATM-mediated phosphorylation events, which signals checkpoint proteins CHK1 and CHK2 to induce cell cycle arrest. It is a critical phase required to signal for DSB repair. Thus, disruption of ATR in human or model organisms hinders DNA repair. We hypothesize that a similar disruption of PRKDC and its vital role in controlling genomic instability would have selective cytotoxic effects in cells with replicative stress induced by MYC. Future studies with *in vivo* models where this PRKDC-MYC connection is perturbed will be essential in order to fully appreciate its clinical importance and relevance.

Aside from its involvement in NHEJ DNA repair, PRKDC has also been implicated in gene regulation and protein abundance. It was reported that RNAi-mediated knockdown of PRKDC decreased the abundance of MYC protein in Hela cells [32]. Mechanistically, phosphorylation of MYC protein on residues Thr58 by GSK3β is essential for its FBW7-mediated proteasomal degradation. Interestingly, it was reported that PRKDC can phosphorylate AKT, which results in the inhibition of GSK3β and subsequent MYC degradation. [32]. Thus, a possible scenario is cancer cells that are ‘addicted’ to MYC become highly sensitive to hindrance of PRKDC function, leading to subsequent interference of MYC transcription and protein translation and cancer cell death (Figure 6).

A number of studies have suggested that PRKDC acts as a modulator of gene transcription. PRKDC phosphorylates a variety of transcription factors including FOS, JUN, SP1, OCT-1, TFIID, E2F, the estrogen receptor, and the large subunit of RNA polymerase II [67, 68]. PRKDC is also thought to be required for transcriptional regulation mediated by transcription factors, such as lymphocyte enhancer factor 1 (LEF1) [69], heat shock transcription factor (HSF) [70], p53 and the product of the predominant ETS gene fusion, TMPRSS2:ERG, in prostate cancer [71]. Future studies should be directed towards investigating whether targeting PRKDC in these settings may have beneficial outcomes.

## Conclusions

Targeting cancer with synthetic lethal partners offers a unique advantage to leave normal tissues unscathed and destroying only the cancer cells. Our study demonstrates that inhibition of PRKDC may offer a therapeutic strategy in MYC-driven cancers.

## Electronic supplementary material

Additional file 1: Table S1: Summary of deep sequencing results for the screen using WI-38 cell lines stably-expressing empty vector (pCDH), L-MYC1 or L-MYC2, after infection with a pooled kinase shRNA library. The read counts were normalized to RPM (Reads per million total reads in the sample; ie (raw reads)/(total reads in the sample) × 10^6^). Minimal requirement for the reads in pCDH-L-MYC1 sample on day 1 is 500. The table is sorted on RPM in pCDH-L-MYC1 sample on day 14. The top nine genes from this table with less than three-fold reduction in control Day1 versus Day14 samples were selected for follow-up. (PDF 682 KB)

Additional file 2: Figure S1: Stable WI-38 cell lines were subject to a cell viability assay at days 1, 3, 5 and 7 and growth curves were analyzed. Data are shown as mean ± SD. Statistical analysis using one-way ANOVA; ****P ≤0.0001; ***P ≤ 0.001; **P ≤ 0.01. (PDF 99 KB)

Additional file 3: Figure S2: Human lymphoma cell lines were divided into two groups, high MYC and low MYC gene expression (BioGPS database values), and treated with PRKDC inhibitors, NU-7441 and KU0060648, for 3 days. Cells were subject to cell viability assays and IC50 of the two PRKDC inhibitors were measured in these cell lines. High MYC and low MYC expression cell lines treated with NU-7441 displayed IC_50_ values of 2.09 + 0.56 μM and 13.76 + 5.18 μM, respectively. The two groups followed a similar trend in terms of IC_50_ when cells were treated with KU0060648 (1.09 + 0.23 μM and 9.27 + 4.71 μM, respectively). Data are shown as mean ± SEM. Statistical analysis using unpaired t test (Mann-Whitney test); **P ≤ 0.01. (PDF 128 KB)

Additional file 4: Figure S3: Different lymphoma cell lines were treated with increasing concentrations of the PRKDC inhibitor, NU-7441, and a proteasome inhibitor, MG132. After a 4 h drug exposure time, c-MYC protein levels were analyzed via immunoblotting with an anti-MYC antibody. PRKDC and GADPH protein levels were also monitored with anti-PRKDC and anti-GADPH antibodies, respectively. (PDF 122 KB)

## Abbreviations

DSB: Double-strand break
PRKDC: Protein Kinase, DNA-Activated, Catalytic Polypeptide
RNAi: RNA interference.
