# Supplementary material for: Identification of synthetic lethality of PRKDC in MYC-dependent human cancers by pooled shRNA screening
Source: BMC Cancer. 2014 Dec 13;14:944. doi: 10.1186/1471-2407-14-944 (PMC4320452; doi:10.1186/1471-2407-14-944)
Supplement: Supplementary file 1 — Additional file 1: Table S1: Summary of deep sequencing results for the screen using WI-38 cell lines stably-expressing empty vector (pCDH), L-MYC1 or L-MYC2, after infection with a pooled kinase shRNA library. The read counts were normalized to RPM (Reads per million total reads in the sample; ie (raw reads)/(total reads in the sample) × 106). Minimal requirement for the reads in pCDH-L-MYC1 sample on day 1 is 500. The table is sorted on RPM in pCDH-L-MYC1 sample on day 14. The top nine genes from this table with less than three-fold reduction in control Day1 versus Day14 samples were selected for follow-up. (PDF 682 KB) [file 12885_2014_5171_MOESM1_ESM.pdf]

		pCDH		pCDH-L-MYC1		pCDH-L-MYC2	
**target**	**Symbol**	**Day1**	**Day14**	**Day1**	**Day14**	**Day1**	**Day14**
NM_006201	CDK16	1078	2013	1072	0	894	561
NM_001798	**CDK2**	826	485	558	0	921	539
NM_001433	ERN1	744	486	794	0	645	1360
NM_002093	**GSK3B**	981	834	773	0	466	726
NM_001080434	LMTK3	892	1330	668	0	591	272
NM_000294	PHKG2	595	1599	530	0	510	483
NM_006254	PRKCD	562	984	932	0	822	279
NM_033126	PSKH2	719	837	788	0	1048	570
NM_012224	NEK1	959	210	1085	0	509	584
NM_001025105	CSNK1A1	591	0	704	0	502	2275
NM_001081640	**PRKDC**	250	842	668	0	444	1402
NM_000906	NPR1	857	292	660	0	723	666
NM_006852	TLK2	711	0	962	0	860	1258
NM_004672	MAP3K6	438	0	858	0	1163	597
NM_003942	RPS6KA4	444	996	605	0	240	605
NM_001522	GUCY2F	538	297	502	0	566	285
NM_007118	TRIO	218	798	553	0	282	0
NM_014791	MELK	283	202	623	0	496	54
NM_003010	MAP2K4	236	79	868	0	191	395
NM_005607	PTK2	226	0	844	0	264	243
NM_014586	HUNK	91	60	687	0	80	48
NM_002227	JAK1	423	0	719	18	544	1236
NM_005990	STK10	1369	1182	1161	19	737	2441
NM_182493	MYLK3	648	545	843	21	847	397
NM_002612	PDK4	233	251	709	22	447	324
NM_004329	BMPR1A	300	758	519	24	276	966
NM_173500	TTBK2	795	55	828	26	439	182
NM_004333	BRAF	932	983	983	30	1277	777
NM_181358	HIPK1	768	344	1004	33	556	384
NM_004935	CDK5	556	395	701	37	465	356
NM_199289	NEK5	506	515	893	51	286	471
NM_152835	PDIK1L	882	427	769	51	477	252
NM_005813	PRKD3	486	1611	671	56	750	36
NM_002019	FLT1	524	210	667	63	710	647
NM_005813	PRKD3	811	749	606	84	949	1250
NM_001699	AXL	869	412	1186	85	833	1021
NM_022048	CSNK1G1	1023	537	1843	89	645	1189
NM_001161565	TNIK	445	900	1485	108	626	521
NM_182398	RPS6KA5	784	1730	1388	123	973	707
NM_016276	SGK2	1237	761	908	128	771	321
NM_000075	CDK4	1432	880	972	134	1388	962
NM_006622	PLK2	980	1009	592	138	699	101
NM_015083	CDK12	966	620	767	143	790	463
NM_139012	MAPK14	932	1035	751	163	784	121
NM_015906	TRIM33	1084	1167	943	165	1048	455

NM_005781	TNK2	169	0	543	167	304	382
NM_022048	CSNK1G1	635	117	673	169	401	626
NM_002740	PRKCI	387	551	652	175	660	218
NM_015148	PASK	640	503	982	177	581	907
NM_001204	BMPR2	1044	1044	657	181	972	1215
NM_001128921	MARK3	1445	1330	1428	186	766	0
NM_001079882	PRKD2	784	1400	568	194	1087	1222
NM_005975	PTK6	555	850	730	197	318	499
NM_006374	STK25	90	302	525	201	434	688
NM_006201	CDK16	581	1359	552	203	1045	1793
NM_001114122	CHEK1	752	1012	1158	207	212	1860
NM_001348	DAPK3	908	722	750	212	1297	485
NM_033141	MAP3K9	626	275	557	215	890	726
NM_002747	MAPK4	337	933	889	216	659	1634
NM_213647	FGFR4	463	385	540	217	768	76
NM_173354	SIK1	576	781	1026	218	593	556
NM_001004057	GRK4	1426	480	1140	226	1009	487
NM_001433	ERN1	273	17	752	228	422	766
NM_031417	MARK4	1051	713	632	233	580	1051
NM_006575	MAP4K5	583	287	1185	238	890	767
NM_006035	CDC42BPB	1110	720	1321	239	1058	943
NM_005160	ADRBK2	280	22	649	246	400	1146
NM_001204	BMPR2	831	1267	505	249	1422	1707
NM_004333	BRAF	501	577	574	259	607	288
NM_002880	RAF1	676	1071	779	259	890	864
NM_003957	BRSK2	913	1293	1253	261	468	766
NM_001111033	ACVR1C	544	165	1459	262	747	805
NM_005204	MAP3K8	1204	1357	1478	266	866	108
NM_005012	ROR1	1266	705	791	266	388	2126
NM_032028	TSSK1B	811	928	1004	267	591	68
NM_004196	CDKL1	826	1294	778	269	621	563
NM_002031	FRK	408	795	1082	270	524	278
NM_000142	FGFR3	573	142	677	270	890	749
NM_015000	STK38L	1469	1167	1487	271	825	1045
NM_014911	AAK1	385	617	599	272	168	81
NM_005356	LCK	1353	552	1034	277	983	1497
NM_006281	STK3	944	556	510	277	491	871
NM_001136138	RPS6KC1	1014	755	897	278	1397	0
NM_213647	FGFR4	845	820	736	282	1024	550
NM_006374	STK25	1799	1002	1486	282	1324	2174
NM_000459	TEK	308	863	559	289	240	205
NM_017525	CDC42BPG	412	545	660	293	339	1336
NM_002497	NEK2	1101	789	1111	294	660	802
NM_018650	MARK1	752	1184	739	298	537	363
NM_007118	TRIO	613	210	917	298	362	1127
NM_172082	CAMK2B	81	105	729	298	150	147
NM_015905	TRIM24	571	396	535	305	833	298

NM_016653	ZAK	791	1740	889	305	858	1252
NM_207519	ZAP70	839	208	675	313	910	483
NM_015906	TRIM33	561	53	711	317	350	127
NM_145185	MAP2K7	1104	1813	597	320	765	821
NM_004441	EPHB1	1300	1265	1237	323	824	1251
NM_001025105	CSNK1A1	837	350	1462	326	1116	1009
NM_006742	PSKH1	657	375	795	329	1187	1171
NM_003993	CLK2	1193	846	678	337	1093	55
NM_139355	MATK	530	559	609	337	297	381
NM_001013703	EIF2AK4	483	432	1242	344	852	162
NM_033266	ERN2	901	1880	1860	349	1560	1550
NM_001018066	NTRK2	960	803	539	350	1349	288
NM_203351	MAP3K3	928	1465	1238	354	1033	422
NM_006852	TLK2	1283	1050	770	355	1685	485
NM_005372	MOS	591	269	737	356	1320	237
NM_006258	PRKG1	473	563	1051	360	776	313
NM_182398	RPS6KA5	1252	225	1250	364	966	749
NM_004073	PLK3	1345	589	510	364	739	221
NM_005592	MUSK	761	18	837	365	465	579
NM_005417	SRC	737	2006	1160	377	726	618
NM_004958	MTOR	1102	794	1202	379	1303	1592
NM_024046	CAMKV	788	1134	918	380	860	147
NM_013233	STK39	711	469	551	380	708	1131
NM_005308	GRK5	1078	2290	1231	384	1627	1333
NM_001142386	PDK3	905	260	1127	385	1185	618
NM_001619	ADRBK1	1258	1908	1062	386	966	378
NM_014840	NUAK1	1106	1010	1075	387	753	1141
NM_004327	BCR	829	725	878	388	1148	890
NM_139355	MATK	1240	898	1132	389	977	1562
NM_007271	STK38	925	1871	696	392	1049	1094
NM_001005735	CHEK2	1505	422	877	395	324	1408
NM_003845	DYRK4	410	102	643	395	949	186
NM_175866	UHMK1	655	981	668	399	843	238
NM_017572	MKNK2	498	907	1125	401	414	682
NM_001896	CSNK2A2	390	124	607	403	295	213
NM_001077401	ACVRL1	643	469	975	407	735	844
NM_033126	PSKH2	533	1060	998	409	585	942
NM_024652	LRRK1	870	197	807	411	826	633
NM_005417	SRC	639	625	527	413	341	404
NM_145686	MAP4K4	613	867	792	415	994	449
NM_001081563	DMPK	1605	1462	1111	416	1376	1666
NM_014226	RAGE	1321	1184	878	418	684	300
NM_001012331	NTRK1	1185	50	1121	421	1068	421
NM_053006	TSSK2	1333	775	951	422	777	2096
NM_003215	TEC	1382	1340	1019	426	1099	3225
NM_002944	ROS1	2239	1843	1961	429	1694	2911
NM_014720	SLK	708	1206	896	430	835	1699

NM_206907	PRKAA1	1463	1392	1821	432	1189	316
NM_002751	MAPK11	1141	1068	1337	436	1368	721
NM_002754	MAPK13	1336	407	1212	439	617	1152
NM_002755	MAP2K1	942	501	612	446	683	31
NM_001106	ACVR2B	1212	549	1243	452	1000	296
NM_015016	MAST3	661	340	747	453	620	1201
NM_139070	MAPK9	1004	203	770	460	677	303
NM_021643	TRIB2	928	809	864	470	1131	523
NM_005211	CSF1R	577	2255	888	471	374	451
NM_002419	MAP3K11	623	217	638	473	186	676
NM_032237	SGK196	392	325	600	475	446	1010
NM_001172303	MASTL	1330	1119	1116	480	1393	633
NM_212502	CDK18	210	0	502	484	66	73
NM_001099439	EPHA10	762	988	804	485	606	983
NM_001799	CDK7	1220	1194	1169	487	1188	775
NM_005781	TNK2	530	991	1074	488	1275	575
XM_496486	GNF087948	479	734	524	489	656	363
NM_031464	RPS6KL1	398	460	532	490	807	164
NM_014602	PIK3R4	1228	741	932	494	803	1000
NM_002595	CDK17	722	749	657	495	525	1457
NM_002576	PAK1	882	972	1210	504	719	641
NM_001111067	ACVR1	665	210	752	504	791	501
NM_001127190	CSK	605	715	1046	505	861	1448
NM_145203	CSNK1A1L	409	417	1298	506	898	52
NM_030952	NUAK2	776	584	737	507	1103	409
NM_006293	TYRO3	1204	1640	1254	508	516	2736
NM_002648	PIM1	427	36	587	511	920	632
NM_001319	CSNK1G2	545	540	625	519	670	1028
NM_004439	EPHA5	814	0	1188	520	853	616
NM_001044722	CSNK1G3	611	806	615	522	1116	694
NM_001048218	SCYL1	287	838	621	522	253	724
NM_001172303	MASTL	166	1168	898	528	643	865
NM_030662	MAP2K2	849	1139	1133	529	913	473
NM_004721	MAP3K13	755	530	920	534	555	1204
NM_001099436	ULK3	618	440	786	535	433	123
NM_173174	PTK2B	657	653	1160	537	777	1190
NM_005308	GRK5	912	620	912	537	870	449
NM_004972	JAK2	1274	546	948	537	834	513
NM_033115	TBCK	990	1884	1881	544	1932	2609
NM_014496	RPS6KA6	812	558	1415	545	861	2188
NM_032538	TTBK1	664	1032	873	545	909	1023
NM_006258	PRKG1	1101	167	663	545	551	1139
NM_001014834	PAK4	1254	144	998	547	1315	1141
NM_004329	BMPR1A	166	598	1156	547	535	797
NM_004329	BMPR1A	1152	632	561	549	469	200
NM_002612	PDK4	532	1096	1238	553	727	671
NM_198578	LRRK2	904	163	887	554	419	755

NM_006213	PHKG1	1400	312	1819	555	862	1776
NM_001040056	MAPK3	775	1194	854	555	434	1494
NM_033266	ERN2	1720	1313	991	557	1212	439
NM_002609	PDGFRB	613	792	686	558	310	454
NM_005232	EPHA1	962	902	901	562	1117	600
NM_171825	CAMK2A	1317	1119	991	566	980	773
NM_004963	GUCY2C	586	472	697	569	757	953
NM_005424	TIE1	451	496	648	572	499	521
NM_033116	NEK9	582	594	514	573	577	212
NM_002929	GRK1	1411	1450	561	574	1671	2453
NM_000180	GUCY2D	727	1814	1066	577	1343	789
NM_172129	CAMK2D	845	1080	559	579	692	1032
NM_001128628	PAK6	692	378	1100	581	778	594
NM_001166171	NEK6	1024	1435	794	590	860	792
NM_001111097	LYN	619	567	675	590	843	985
NM_004439	EPHA5	1413	4237	982	598	1258	2637
NM_005465	AKT3	272	91	773	601	480	265
NM_001042452	RP6-213H19.1	1722	2417	1179	605	2079	1637
NM_003318	TTK	659	841	865	607	422	448
NM_001162407	CLK1	625	1174	552	609	1122	566
NM_004431	EPHA2	922	617	1936	612	807	550
NM_002755	MAP2K1	1318	1108	1601	614	930	695
NM_001081640	PRKDC	1603	1495	2481	622	2004	1164
NM_052841	TSSK3	740	287	568	623	630	883
NM_001619	ADRBK1	1077	465	947	624	777	590
NM_139209	GRK7	942	1235	768	627	938	654
NM_003157	NEK4	423	887	814	628	427	1095
NM_032435	KIAA1804	732	677	905	629	877	1650
NM_021135	RPS6KA2	1070	737	863	630	1074	817
NM_032960	MAPKAPK2	746	298	702	634	1042	1317
NM_001081640	PRKDC	842	627	837	635	1472	983
NM_145109	MAP2K3	344	1460	1335	636	667	452
NM_003957	BRSK2	341	322	505	638	495	1124
NM_145331	MAP3K7	359	1151	1347	640	612	398
NM_005157	ABL1	780	812	859	640	467	382
NM_182493	MYLK3	1171	869	1249	641	1017	741
NM_021158	TRIB3	1250	1142	1268	643	1507	1710
NM_138370	PKDCC	305	897	504	645	587	651
NM_001166695	SLC1A3	788	276	904	645	473	731
NM_030662	MAP2K2	536	1302	602	648	662	122
NM_003160	AURKC	369	569	515	649	568	1008
NM_002419	MAP3K11	1511	1469	1152	654	1061	791
NM_021133	RNASEL	1225	1725	1001	655	1297	2067
NM_012290	TLK1	992	771	1141	655	771	971
NM_145331	MAP3K7	1580	540	2082	656	1600	647
NM_006285	TESK1	935	130	709	658	705	769
NM_031965	GSG2	1529	620	998	661	955	789

NM_022048	CSNK1G1	1129	2353	1251	666	1963	1578
NM_001166171	NEK6	862	775	850	668	856	658
NM_002611	PDK2	734	393	951	669	1128	1399
NM_001184	ATR	747	386	554	670	1139	1009
NM_006875	PIM2	493	0	694	672	336	65
NM_001170761	SRPK3	1393	164	851	673	998	1862
NM_152835	PDIK1L	1257	828	1190	677	1430	267
NM_003496	TRRAP	1518	2120	1668	678	1490	2505
NM_002748	MAPK6	1263	1576	1669	679	1893	1372
NM_001113239	HIPK2	1400	1133	1115	684	1707	523
NM_014975	MAST1	961	1335	1120	684	863	1209
NM_003565	ULK1	1504	1621	1432	686	1608	979
NM_153047	FYN	1500	343	669	692	793	988
NM_007271	STK38	1276	1074	1629	694	1528	814
NM_014572	LATS2	898	1369	648	694	774	605
NM_016281	TAOK3	2181	2261	2723	699	1827	2592
NM_031464	RPS6KL1	625	2502	1539	700	1146	1545
NM_000875	IGF1R	751	682	844	704	766	68
NM_005906	MAK	877	1028	706	709	551	274
NM_002497	NEK2	1161	660	1213	710	627	324
NM_145109	MAP2K3	577	279	1056	710	772	979
NM_001799	CDK7	1015	773	777	715	1171	859
NM_000459	TEK	1450	773	1809	718	963	986
NM_004560	ROR2	1037	850	561	723	708	439
NM_002754	MAPK13	870	763	759	723	1063	574
NM_182691	SRPK2	390	691	650	724	834	1097
NM_015083	CDK12	995	1011	1419	725	798	1127
NM_001099436	ULK3	603	438	756	728	579	810
NM_020439	CAMK1G	677	906	634	735	512	861
NM_001111067	ACVR1	1380	744	1411	738	1509	908
NM_016653	ZAK	1264	1339	1169	738	751	2051
NM_003384	VRK1	1081	1967	1074	738	963	548
NM_001111097	LYN	669	1238	1160	739	1190	1322
NM_006241	PPP1R2	1527	1705	1702	740	1674	2311
NM_001005862	ERBB2	578	420	968	743	718	641
NM_001259	CDK6	513	276	708	743	593	1001
NM_001165969	STRADA	706	955	639	745	697	724
NM_002031	FRK	1379	648	620	746	1727	1013
NM_001160367	CDK10	751	1363	1459	746	1103	706
NM_003821	RIPK2	494	255	592	747	195	810
NM_003954	MAP3K14	1112	2128	1855	749	1719	2073
NM_004635	MAPKAPK3	577	553	757	750	609	1124
NM_001032296	STK24	1474	1225	1070	752	723	1808
NM_053006	TSSK2	742	1527	1118	753	933	479
NM_002227	JAK1	981	691	866	754	618	605
NM_005592	MUSK	953	138	937	755	755	1176
NM_001122957	BCKDK	760	566	1065	758	453	73

NM_032037	TSSK6	1186	346	701	761	1062	442
NM_015083	CDK12	1420	887	843	761	449	864
NM_182925	FLT4	574	25	767	761	671	671
NM_020975	RET	729	491	835	762	254	0
NM_020639	RIPK4	1175	229	579	764	704	816
NM_007199	IRAK3	1663	1578	1905	767	1421	2511
NM_005921	MAP3K1	1092	971	1584	769	905	188
NM_004938	DAPK1	373	1822	595	769	607	645
NM_014586	HUNK	847	1002	1303	771	773	1441
NM_013254	TBK1	1768	1152	1856	772	1829	511
NM_001170761	SRPK3	1017	662	775	780	683	1403
NM_172129	CAMK2D	1096	618	1814	782	1045	453
NM_012395	CDK14	1948	622	1345	786	2358	512
NM_001203	BMPR1B	1308	1983	906	788	622	1713
NM_139021	MAPK15	940	1010	711	788	826	1170
NM_003496	TRRAP	908	287	1462	789	585	495
NM_002880	RAF1	518	1144	702	790	902	1152
NM_198465	NRK	1366	2564	803	795	1730	1692
NM_005308	GRK5	414	17	793	796	543	910
NM_005921	MAP3K1	815	168	777	797	863	424
NM_002880	RAF1	1561	1442	2255	799	762	1128
NM_004972	JAK2	1507	1115	1409	799	1648	387
NM_020341	PAK7	1524	479	1781	800	1033	71
NM_004963	GUCY2C	774	1215	1071	808	1087	1020
NM_004439	EPHA5	1146	615	877	810	1239	1268
NM_002613	PDPK1	911	952	849	811	457	645
NM_014791	MELK	1157	966	1312	812	922	905
NM_139034	MAPK7	279	228	655	812	396	986
NM_017572	MKNK2	923	276	1052	813	1582	942
NM_145109	MAP2K3	911	1258	1166	815	650	825
NR_026557	PLK5P	710	1442	1600	827	802	84
NM_138995	MYO3B	301	109	853	828	458	653
NM_001114182	IRAK4	312	325	508	831	284	527
NM_172082	CAMK2B	1177	586	800	832	1153	1128
NM_182398	RPS6KA5	3072	2945	2273	836	1581	821
NM_003010	MAP2K4	136	34	679	838	411	0
NM_001009565	CDKL4	400	829	509	839	306	997
NM_015112	MAST2	853	1464	1032	845	772	451
NM_015905	TRIM24	1062	874	560	846	741	1087
NM_015092	SMG1	1904	1711	1278	858	1536	850
NM_015112	MAST2	1939	973	1416	860	1283	990
NM_000245	MET	486	103	1013	860	1307	1437
NM_018650	MARK1	833	52	701	860	756	1000
NM_001168238	ABL2	1027	585	1288	861	1143	207
NM_018650	MARK1	952	1465	675	870	1466	704
NM_001001671	MAP3K15	1138	717	1253	870	951	0
NM_032017	STK40	521	762	1000	876	661	1468

NM_003993	CLK2	439	1100	1260	877	780	169
NM_002741	PKN1	1166	1113	1003	881	763	639
NM_006871	RIPK3	910	1534	1322	882	997	285
NM_181093	SCYL3	761	228	863	885	522	576
NM_020639	RIPK4	1127	1234	1986	886	1139	1572
NM_003159	CDKL5	789	963	1120	888	480	1038
NM_001042599	ERBB4	1815	126	1481	892	857	647
NM_139047	MAPK8	904	1583	1461	893	876	986
NM_182687	PKMYT1	912	1102	1200	894	971	2053
NM_033116	NEK9	881	989	1266	894	614	1588
NM_207519	ZAP70	296	1155	954	898	962	203
NM_004444	EPHB4	1052	941	1595	902	1408	1201
NM_024776	SGK269	1027	768	2059	902	921	1314
NM_019884	GSK3A	1316	1994	1569	904	1544	1028
NM_145001	STK32A	926	2389	1876	904	1293	1640
NM_003952	RPS6KB2	1284	1033	981	905	1886	1395
NM_033116	NEK9	604	376	686	906	1184	1389
NM_017672	TRPM7	803	648	1171	908	1958	1877
NM_003913	PRPF4B	1422	1403	1484	910	1349	1311
NM_014975	MAST1	1123	899	656	910	955	1110
NM_001012418	MYLK4	1163	2262	1345	911	1106	971
NM_004196	CDKL1	999	924	956	911	924	612
NM_031965	GSG2	978	994	1360	914	768	908
NM_000061	BTK	553	941	916	915	735	459
NM_212502	CDK18	603	18	585	915	951	605
NM_020439	CAMK1G	1035	1191	924	916	774	1326
NM_018979	WNK1	764	109	1392	918	1069	1236
NM_006575	MAP4K5	931	1022	993	919	455	973
NM_004635	MAPKAPK3	1622	1899	846	920	1043	1275
NM_003993	CLK2	849	1202	1971	923	1041	1169
NM_007170	TESK2	1362	1328	1490	926	760	169
NM_080823	SRMS	867	482	1259	929	891	932
NM_006648	WNK2	884	640	1142	936	666	929
NM_006875	PIM2	532	589	580	940	823	900
NM_001170639	CDK20	996	569	592	941	847	1874
NM_002610	PDK1	504	1048	866	949	760	653
NM_002969	MAPK12	799	63	906	949	799	0
NM_015191	SIK2	942	12	1678	953	864	1359
NM_001109891	MAPK3	1156	212	2130	954	979	1414
NM_020328	ACVR1B	1831	935	2192	955	1582	1208
NM_014215	INSRR	1490	1173	1982	957	1663	1315
NM_198892	BMP2K	831	1115	1004	958	874	1127
NM_004443	EPHB3	1219	397	918	963	792	1367
NM_033403	DCLK3	1351	800	1502	965	1062	1073
NM_001018066	NTRK2	654	1543	752	970	940	1346
NM_002419	MAP3K11	903	902	1106	973	988	250
NM_170709	SGK3	746	18	605	978	188	1260

NM_001556	IKBKB	436	1091	1031	979	267	1427
NM_080836	STK35	1209	2179	1502	982	1301	1932
NM_182925	FLT4	898	632	595	985	857	706
NR_028062	PRKY	1068	1188	766	990	823	525
NM_207518	PRKACA	660	1232	673	990	1259	453
NM_032960	MAPKAPK2	1158	650	1558	993	2121	1204
NM_207578	PRKACB	1691	565	829	994	1768	502
NM_003957	BRSK2	972	608	1088	996	1216	2182
NM_001893	CSNK1D	945	614	877	996	486	1337
NM_005372	MOS	1412	809	1326	998	1193	1045
NM_175866	UHMK1	897	318	748	998	782	1025
NM_001032296	STK24	1118	813	622	1001	616	225
NM_001077401	ACVRL1	1194	926	1277	1003	1643	3190
NM_005372	MOS	1490	1026	520	1003	1096	718
NM_001142610	ULK2	1118	2202	860	1016	1324	1879
NM_013392	NRBP1	398	446	759	1016	1265	581
NM_003331	TYK2	1055	1195	1776	1017	919	1459
NM_001001852	PIM3	854	727	734	1019	803	1321
NM_001128628	PAK6	559	381	1019	1026	1087	1171
NM_003582	DYRK3	1699	1347	695	1032	1602	2156
NM_020791	TAOK1	712	798	1198	1032	1371	888
NM_172129	CAMK2D	1605	2194	1463	1039	1501	1099
NM_016276	SGK2	703	531	971	1039	982	926
NM_001744	CAMK4	1626	548	1103	1040	849	582
NM_002821	PTK7	927	1579	1266	1044	853	1483
NM_021643	TRIB2	772	446	1358	1045	693	0
NM_153498	CAMK1D	1217	579	1575	1047	1690	1059
NM_139047	MAPK8	477	943	1189	1053	931	1395
NM_001570	IRAK2	931	754	1593	1055	1035	213
NM_001081563	DMPK	587	1255	822	1056	968	328
NM_001654	ARAF	371	954	774	1056	381	1460
NM_002577	PAK2	1229	1959	2076	1058	910	578
NM_017662	TRPM6	1497	2103	967	1058	1113	1161
NM_002610	PDK1	557	2720	891	1058	539	1065
NM_173575	STK32C	1021	664	1018	1058	1327	1427
NM_002446	MAP3K10	966	284	1250	1063	1286	407
NM_003242	TGFBR2	2939	2144	1455	1064	2519	2772
NM_001278	CHUK	805	1466	1043	1065	711	715
NM_007170	TESK2	637	1095	1202	1067	956	1013
NM_025195	TRIB1	814	941	958	1070	1228	1891
NM_015690	STK36	393	935	677	1072	785	682
NM_030906	STK33	1347	578	728	1077	494	515
NM_001048200	HIPK3	832	1342	1010	1078	733	1461
NM_000875	IGF1R	555	417	802	1081	621	61
NM_001014795	ILK	372	680	829	1082	601	472
NM_030952	NUAK2	1520	1835	2334	1089	1507	1208
NM_020666	CLK4	406	272	774	1092	733	1083

NM_004336	BUB1	1799	1634	2243	1103	2148	1300
NM_080836	STK35	832	936	772	1105	1192	1073
NM_016440	VRK3	902	215	672	1105	1094	968
NM_001113239	HIPK2	2006	1468	1112	1112	1350	1841
NM_001172131	HCK	1088	1152	795	1112	979	493
NM_021135	RPS6KA2	776	332	678	1113	1355	566
NM_198892	BMP2K	1543	1051	1379	1115	1128	481
NM_006575	MAP4K5	506	617	1107	1124	610	266
NM_004226	STK17B	773	615	1194	1125	680	717
NM_014002	IKBKE	1185	95	1613	1127	1091	895
NM_003159	CDKL5	999	1458	986	1127	809	725
NM_001222	CAMK2G	1051	207	812	1129	574	351
NM_003656	CAMK1	580	436	543	1130	915	1720
NM_001128173	PAK3	901	1441	1064	1131	764	928
NM_013233	STK39	679	231	668	1131	484	833
NM_001135740	PNCK	1295	890	1593	1132	1444	1977
NM_004440	EPHA7	1694	510	2113	1137	674	1296
NM_199289	NEK5	585	891	963	1139	411	514
NM_001005915	ERBB3	1200	1178	1563	1158	1783	1028
XM_067723	GNF090716	576	302	1180	1159	704	503
NM_001040261	DCLK2	555	1221	641	1160	741	702
NM_002758	MAP2K6	1275	2619	1212	1161	930	922
NM_001008910	STK16	1382	728	1399	1164	1034	912
NM_006285	TESK1	925	1202	1200	1169	1229	743
NM_005228	EGFR	851	935	2234	1170	1392	1557
NM_031480	RIOK1	2369	479	1057	1175	2155	822
NM_007118	TRIO	1076	1741	809	1176	1469	1645
NM_153498	CAMK1D	499	1087	873	1179	926	1104
NM_001044722	CSNK1G3	979	728	2087	1181	571	1099
NM_004734	DCLK1	1142	954	816	1181	1516	997
NM_001113239	HIPK2	938	1289	967	1181	983	448
NM_153499	CAMKK2	1076	425	1338	1182	1851	760
NM_002929	GRK1	1131	1067	1292	1183	996	1425
NM_053006	TSSK2	826	362	948	1184	766	1716
NM_139078	MAPKAPK5	1378	1231	1887	1185	1783	1281
NM_003582	DYRK3	961	1288	1872	1185	1453	1914
NM_001142386	PDK3	455	11	718	1185	403	423
NM_033493	CDK11B	1210	1949	764	1192	1564	3653
NM_005211	CSF1R	1182	1321	899	1194	1041	783
NM_021133	RNASEL	573	924	819	1199	775	894
NM_032538	TTBK1	124	615	797	1199	304	385
NM_032387	WNK4	811	628	959	1202	633	1085
NM_003948	CDKL2	834	2301	2027	1205	1926	1414
NM_015148	PASK	1739	1984	1667	1209	1754	832
NM_003157	NEK4	503	146	530	1210	625	604
NM_031267	CDK13	998	1538	568	1212	541	817
NM_015690	STK36	1824	1446	1687	1216	2790	1801

NM_001018046	YSK4	1065	2250	992	1224	1735	2417
NM_001098623	OBSCN	2216	1651	2166	1225	1519	1329
NM_002648	PIM1	1064	920	1302	1228	1249	765
NM_006724	MAP3K4	1451	41	640	1232	1221	593
NM_004073	PLK3	794	1259	872	1234	1282	1366
NM_004938	DAPK1	2030	2559	1656	1237	1366	2209
NM_001012331	NTRK1	1809	1774	2443	1237	1822	828
NM_080823	SRMS	576	959	782	1239	597	1064
NM_001259	CDK6	882	86	1050	1239	880	545
NM_005204	MAP3K8	1726	1041	1547	1246	943	2078
NM_001009565	CDKL4	1389	1608	2667	1261	1476	632
NM_000142	FGFR3	822	1110	2356	1267	1419	1927
NM_018423	STYK1	799	1021	1080	1267	1088	143
NM_005546	ITK	1376	1504	1307	1270	2123	1475
NM_014586	HUNK	1118	1995	2107	1279	809	1358
NM_031267	CDK13	428	1453	1758	1279	1316	899
NM_001433	ERN1	1840	1831	1924	1282	2149	1341
NM_002958	RYK	1994	880	2060	1288	927	1164
NM_022965	FGFR3	528	874	804	1288	572	360
NM_144685	HIPK4	1439	1601	1382	1289	1122	859
NM_032037	TSSK6	1815	1547	1594	1290	2380	1663
NM_003384	VRK1	424	439	558	1290	901	959
NM_182644	EPHA3	813	233	504	1290	447	29
NM_006258	PRKG1	1327	1965	1994	1293	711	1653
NM_001012338	NTRK3	1685	1935	1816	1299	1749	1524
NM_002737	PRKCA	2209	1135	1901	1299	1619	1348
NM_032028	TSSK1B	269	1106	942	1301	241	726
NM_006609	MAP3K2	1893	2152	974	1313	1802	1932
NM_025164	SIK3	1395	1968	1423	1313	2320	640
NM_001048218	SCYL1	976	1200	1406	1318	1299	334
NM_002610	PDK1	366	592	519	1336	1134	0
NM_014264	PLK4	1312	2890	1152	1339	1982	1650
NM_015183	MAST4	2022	2132	1108	1341	1168	651
NM_002739	PRKCG	614	395	862	1347	1500	871
NM_017662	TRPM6	591	902	1162	1355	697	1645
NM_016276	SGK2	1129	1492	1228	1357	804	922
NM_138292	ATM	788	589	550	1367	406	1783
NM_015183	MAST4	1144	1177	1278	1371	792	1328
NM_007174	CIT	1838	1653	1726	1374	1440	861
NM_207578	PRKACB	756	932	1259	1374	811	348
NM_001014795	ILK	681	427	1693	1376	465	557
NM_003913	PRPF4B	1502	1325	1203	1377	2066	1473
NM_002821	PTK7	1872	1392	1397	1377	1553	1463
NM_003318	TTK	1427	977	1286	1383	576	538
NM_002609	PDGFRB	1392	1742	1795	1387	1723	1594
NM_005627	SGK1	1203	946	1552	1389	1439	1930
NM_006293	TYRO3	1028	977	1004	1392	1193	1484

NM_033403	DCLK3	704	890	1373	1395	877	366
NM_006259	PRKG2	915	669	582	1403	627	1003
NM_178564	NRBP2	1264	1314	994	1405	1214	1808
NM_016231	NLK	1561	1104	1259	1412	2045	843
NM_001024401	SBK1	505	311	1519	1414	747	1453
NM_139070	MAPK9	1306	3421	1921	1416	1326	1136
NM_152534	GNF084368	387	0	1220	1418	553	940
NM_001626	AKT2	1163	1254	796	1418	1404	375
NM_005417	SRC	1285	966	1262	1419	1334	1164
NM_015092	SMG1	1289	1495	1272	1421	1558	1354
NM_152720	NEK3	993	1294	833	1421	666	1304
NM_005569	LIMK2	1179	1380	1532	1426	1037	949
NM_003821	RIPK2	1836	3041	1180	1426	2539	2112
NM_002031	FRK	1632	737	1200	1427	1511	823
NM_015191	SIK2	1496	1617	784	1439	1435	1117
NM_015016	MAST3	1367	1372	1642	1442	1296	1159
NM_002745	MAPK1	855	464	1784	1442	753	988
NM_006622	PLK2	1521	1256	3031	1447	1869	1530
NM_007174	CIT	1449	1486	1314	1447	1572	2101
NM_001896	CSNK2A2	1340	1712	1039	1447	1321	321
NM_033493	CDK11B	1335	752	1360	1451	668	1058
NM_145686	MAP4K4	1786	2229	1397	1452	2121	997
NM_014572	LATS2	1406	1561	1615	1461	2218	1804
NM_001128921	MARK3	1459	922	1086	1463	1834	1061
NM_025195	TRIB1	604	471	522	1465	380	711
NM_002958	RYK	1127	1244	1847	1465	877	2469
NM_133494	NEK7	1303	1691	972	1477	361	876
NM_012290	TLK1	1197	112	1195	1480	1168	666
NM_203351	MAP3K3	2052	1394	1976	1484	1246	1974
NM_145001	STK32A	853	1565	1195	1486	801	506
NM_003607	CDC42BPA	705	2079	804	1488	887	1220
NM_014496	RPS6KA6	2173	1686	1745	1490	2398	2440
NM_001079882	PRKD2	1007	185	997	1491	606	326
NM_005406	ROCK1	896	1072	865	1503	707	859
NM_004612	TGFBR1	1755	1090	1213	1513	762	968
NM_020630	RET	843	1281	1562	1514	876	1207
NM_003010	MAP2K4	695	865	626	1517	520	412
NM_001128921	MARK3	1128	1912	1149	1518	979	1298
NM_003160	AURKC	2026	970	1203	1530	1879	896
NM_015375	DSTYK	2329	1396	2013	1539	1315	1640
NM_198578	LRRK2	1241	885	1946	1543	1439	1137
XR_037003	LOC283155	1047	972	1481	1544	946	48
NM_015076	CDK19	1065	1295	1378	1549	1269	1071
NM_005246	FER	879	770	1271	1550	1548	620
NM_001136138	RPS6KC1	1227	847	1530	1551	841	1119
NM_001135740	PNCK	1682	767	887	1558	1546	575
NM_015112	MAST2	559	497	748	1574	761	392

NM_182687	PKMYT1	977	353	1225	1578	684	910
NM_032960	MAPKAPK2	2170	2133	2990	1581	2610	2303
NM_001112808	TNNI3K	1337	1084	570	1584	1125	1210
NM_032028	TSSK1B	1719	251	1037	1589	684	928
NM_004734	DCLK1	792	648	1604	1592	930	1228
NM_004579	MAP4K2	2272	3163	3011	1598	3556	2589
NM_002613	PDPK1	2262	2952	2437	1600	2794	1965
NM_001222	CAMK2G	2004	2119	1722	1617	2365	1571
NM_181093	SCYL3	1489	709	1378	1620	1484	3213
NM_005906	MAK	1794	2974	521	1630	1686	1232
NM_014226	RAGE	965	1584	1505	1631	1141	1794
NM_001136138	RPS6KC1	1070	501	1138	1633	610	787
NM_002611	PDK2	1477	1865	1981	1636	1911	2033
NM_001261	CDK9	2012	1819	1558	1639	1623	883
NM_005569	LIMK2	842	435	1040	1642	1028	321
NM_015076	CDK19	1784	2398	782	1648	2126	2095
NM_001799	CDK7	703	1167	927	1648	617	1070
NM_001113575	CDKL3	890	631	724	1651	692	1231
NM_172206	CAMKK1	1217	1590	1568	1655	2277	856
NM_033493	CDK11B	619	593	1408	1655	870	502
NM_177560	CSNK2A1	1088	675	736	1672	921	864
NM_002742	PRKD1	672	666	1306	1680	913	1264
NM_001203	BMPR1B	992	467	577	1682	484	296
NM_001048200	HIPK3	700	1435	846	1684	1393	644
NM_021133	RNASEL	1452	843	1536	1696	1798	2383
NM_152649	MLKL	1657	982	1546	1698	1103	1294
NM_017771	PXK	1670	1138	1321	1714	1716	2131
NM_001954	DDR1	1778	1323	1365	1739	1589	1455
NM_032538	TTBK1	2481	2691	1861	1743	1926	4181
NM_033118	MYLK2	1722	3622	2969	1743	1475	1195
NM_003942	RPS6KA4	1464	1613	1437	1745	1618	868
NM_001005735	CHEK2	1713	2551	1530	1759	2320	2054
NM_178170	NEK8	1480	1870	2495	1769	1082	1721
NM_000208	INSR	1627	1657	1878	1774	1562	2625
NM_212535	PRKCB	2025	1394	1800	1774	1776	633
NM_052841	TSSK3	1904	1893	1287	1778	1426	1110
NM_031464	RPS6KL1	1092	1636	764	1780	1304	513
NM_021135	RPS6KA2	1089	722	1857	1784	1727	1130
NM_005160	ADRBK2	818	1200	2190	1789	1138	1016
NM_004690	LATS1	960	679	1265	1793	996	1512
NM_004721	MAP3K13	666	1592	777	1797	677	1673
NM_005157	ABL1	2122	3526	2114	1798	2082	1674
NM_002595	CDK17	924	2551	697	1799	1164	2541
NM_003845	DYRK4	1846	2176	1928	1802	2683	1225
NM_020630	RET	1567	2131	1259	1809	2340	1365
NM_032960	MAPKAPK2	1599	1371	1933	1814	1608	2290
NM_012224	NEK1	1868	1810	1693	1817	1266	2608

NM_019884	GSK3A	2874	1806	3271	1844	2686	2488
NM_153498	CAMK1D	1080	1792	749	1844	1557	1756
NM_003913	PRPF4B	1179	1485	1880	1848	1223	1077
NM_003503	CDC7	2214	1926	1226	1863	1379	1300
NM_004217	AURKB	1231	657	1366	1872	1069	1118
NM_015905	TRIM24	1399	1342	1859	1874	1291	2544
NM_004690	LATS1	1183	531	1426	1874	1224	755
NM_005465	AKT3	1235	1772	1675	1877	1544	1844
NM_198268	HIPK1	3113	3018	2888	1883	3016	3802
NM_001006665	RPS6KA1	606	632	1629	1884	530	1287
NM_001721	BMX	1299	1377	1000	1893	1236	2320
NM_001042600	MAP4K1	2135	2508	1551	1894	1544	1127
NM_004119	FLT3	1773	1390	2073	1896	1171	238
NM_002093	GSK3B	960	1614	1464	1912	1590	1643
NM_001164690	AMHR2	1536	1629	829	1939	1213	2161
NM_017672	TRPM7	440	1259	966	1945	1119	0
NM_002595	CDK17	1218	916	1505	1948	1393	2158
NM_001715	BLK	1464	1138	623	1953	902	906
NM_139012	MAPK14	853	1382	1702	1959	985	751
NM_003215	TEC	1121	1309	2042	1976	1254	1220
NM_031267	CDK13	1957	1187	1701	1978	1621	1316
NM_001163297	MARK2	1635	1098	1379	1979	1139	2788
NM_133494	NEK7	729	260	1102	1979	792	1389
NM_001004106	GRK6	1610	2390	1133	1989	1146	881
NM_001172645	TK2	1964	2370	1721	2007	1684	2012
NM_001699	AXL	1344	1906	1460	2009	1081	651
NM_007174	CIT	1251	1065	992	2012	1603	701
NM_005781	TNK2	1283	2755	1519	2022	1459	892
NM_001113575	CDKL3	1850	1543	771	2067	890	732
NM_016653	ZAK	2199	1479	948	2083	1599	1689
NM_139034	MAPK7	1965	1460	1240	2085	2902	2386
NM_018571	STRADB	2042	2296	713	2086	1582	1294
NM_004612	TGFBR1	2721	2202	2270	2087	2245	1596
NM_020791	TAOK1	1721	100	1069	2089	727	995
NM_000455	STK11	1272	3319	2101	2090	1519	1001
NM_170663	MINK1	1722	2290	1393	2093	1745	1451
NM_145160	MAP2K5	1032	497	1973	2102	873	660
NM_003618	MAP4K3	1339	608	764	2112	1006	787
NM_182644	EPHA3	1320	2381	1314	2122	2105	1830
NM_006254	PRKCD	2106	1725	1849	2127	2281	626
NM_006343	MERTK	1700	3813	2215	2134	2041	2314
NM_032387	WNK4	1106	630	844	2141	756	599
NM_138292	ATM	1632	502	1794	2151	1282	1921
NM_004440	EPHA7	1732	2949	774	2157	1795	2019
NM_139078	MAPKAPK5	1296	1713	1254	2157	1722	1508
NM_001211	BUB1B	1263	509	1207	2173	814	1666
NM_031480	RIOK1	827	1706	1520	2176	755	1364

NM_033126	PSKH2	2782	1867	2562	2185	3347	3012
NM_152720	NEK3	1791	582	2538	2188	1622	2879
NM_207578	PRKACB	2997	3932	2862	2199	2177	2794
NM_005109	OXSR1	976	1619	1369	2234	1016	1767
NM_000455	STK11	1244	947	1077	2246	954	1104
NM_003995	NPR2	1938	1822	1921	2248	1936	986
NM_001014796	DDR2	3478	4649	3320	2250	3493	2186
NM_004734	DCLK1	893	1733	544	2254	647	735
NM_004958	MTOR	2046	2470	2001	2270	2120	1196
NM_000061	BTK	1695	1034	1077	2278	1204	833
NM_001184	ATR	1567	2703	1839	2297	1954	1528
NM_014326	DAPK2	2135	3218	3101	2301	3604	3326
NM_005012	ROR1	1564	1245	1752	2309	1652	1653
NM_002944	ROS1	2023	1133	1387	2313	2217	1719
NM_003177	SYK	1722	3987	2908	2319	1980	1460
NM_001100594	SNRK	685	974	628	2319	794	733
NM_001556	IKBKB	1385	3703	1898	2327	1394	1094
NM_153047	FYN	1570	330	1717	2351	1683	1443
NM_198465	NRK	2749	2461	3254	2361	3242	4203
NM_006213	PHKG1	1616	1828	1172	2387	1486	1794
NM_003565	ULK1	1472	1797	866	2389	1998	1188
NM_001024401	SBK1	1935	1250	2186	2391	1929	1215
NM_001004106	GRK6	714	2506	2230	2468	1368	1938
NM_005813	PRKD3	2716	2204	2591	2489	2616	4108
NM_006875	PIM2	2196	1822	1887	2498	1661	471
NM_139158	CDK15	1080	1024	1855	2503	1058	1143
NM_006343	MERTK	1772	3071	1407	2560	1955	2116
NM_001025105	CSNK1A1	1619	1298	1057	2564	1452	2447
NM_017988	SCYL2	3171	2408	2389	2598	2323	1728
NM_004336	BUB1	1605	1818	1749	2600	1451	2156
NM_003159	CDKL5	2052	1918	1274	2608	1147	2362
NM_032435	KIAA1804	1696	1148	1901	2614	1197	1292
NM_000294	PHKG2	1589	1838	1923	2619	2277	1260
NM_006301	MAP3K12	3057	1853	1930	2622	1942	2674
NM_001122833	STK31	613	614	546	2622	1552	1095
NM_030662	MAP2K2	2717	2451	2074	2636	3421	3796
NM_001004057	GRK4	2367	778	1487	2661	1251	2713
NM_001135685	LTK	2269	2031	1990	2684	2079	2186
NM_002741	PKN1	1866	3338	2336	2692	2099	2390
NM_004438	EPHA4	2801	3948	1731	2718	2677	3858
NM_005400	PRKCE	2043	2638	1938	2727	1832	1797
NM_003242	TGFBR2	3291	3415	2330	2748	2775	2426
NM_001009565	CDKL4	1152	2190	1336	2748	1668	737
NM_001100594	SNRK	1610	712	1727	2790	1864	821
NM_173500	TTBK2	1829	2009	2548	2808	2041	3565
NM_145001	STK32A	2869	1120	2173	2818	2254	2918
NM_032430	BRSK1	950	2155	1276	2820	1482	1270

NM_001721	BMX	3088	2827	2206	2848	2357	1873
NM_005204	MAP3K8	1526	1496	1259	2895	1715	1697
NM_001004057	GRK4	1831	1384	774	2899	1576	1801
NM_001128628	PAK6	1459	1215	1779	2916	1924	1574
NM_013254	TBK1	2894	2890	1932	2921	2398	3766
NM_006609	MAP3K2	990	1945	2533	2929	1362	1872
NM_005109	OXSR1	2250	1525	2538	2940	1453	2390
NM_004935	CDK5	1204	3008	1295	2956	2267	3053
NM_002821	PTK7	2615	2947	2445	2983	2738	1233
NM_004327	BCR	1748	739	1432	2998	1335	2220
NM_032237	SGK196	3072	2282	1659	3102	2184	2174
NM_002497	NEK2	3093	3673	3126	3147	2594	2633
NM_018979	WNK1	2735	3769	3193	3218	2083	5554
NM_000294	PHKG2	2782	2845	2378	3219	2730	1612
NM_004963	GUCY2C	1398	2467	1465	3244	2114	2525
NM_001005862	ERBB2	1517	2063	1536	3255	1970	2055
NM_006301	MAP3K12	1058	1868	2389	3337	1620	2308
NM_001143784	FES	1548	3020	1546	3367	2399	2377
NM_138980	MAPK10	1617	2680	1652	3397	1792	1282
NM_031417	MARK4	1438	2807	2405	3417	1556	1745
NM_001013703	EIF2AK4	1964	2749	1848	3423	1499	1039
NM_001348	DAPK3	1580	1161	1286	3460	2068	2478
NM_080823	SRMS	2841	2390	2573	3512	2052	2451
NM_004579	MAP4K2	905	909	1864	3514	1580	1331
NM_207518	PRKACA	2650	3333	3190	3568	2235	2024
NM_139209	GRK7	2284	2845	2476	3598	3224	1783
NM_023108	FGFR1	2291	2165	2517	3702	2135	4860
NM_001744	CAMK4	4260	3155	2355	3714	4128	2976
NM_133494	NEK7	1846	1634	2143	3760	2116	2085
NM_152221	CSNK1E	2128	1204	3250	3795	2180	3458
NM_017525	CDC42BPG	1419	1788	1199	3819	1758	3063
NM_001142386	PDK3	1657	1130	1669	3948	1280	1409
NM_001143976	WEE1	1410	2431	1518	4272	1581	1764
NM_001079882	PRKD2	1935	2732	1974	4355	1880	2697
NM_001040261	DCLK2	2271	2951	1189	4375	1682	2526
NM_002969	MAPK12	2449	3490	3529	4446	2875	3449
NM_015375	DSTYK	5810	4431	3538	4450	4496	9366
NM_001126054	CASK	5545	5900	4201	4495	4636	5622
NM_002612	PDK4	4113	2028	3818	4768	5217	5085
NM_005465	AKT3	1386	3276	2439	5101	1817	2133
NM_004938	DAPK1	3224	3657	1938	6140	3676	3875
