# Supplementary figures and images for: Identification of synthetic lethality of PRKDC in MYC-dependent human cancers by pooled shRNA screening
Source: BMC Cancer. 2014 Dec 13;14:944. doi: 10.1186/1471-2407-14-944 (PMC4320452; doi:10.1186/1471-2407-14-944)

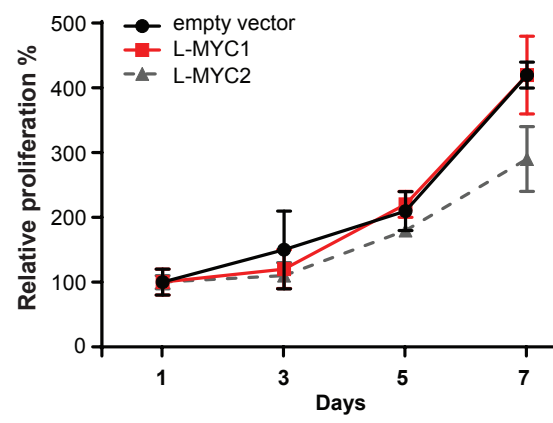

Supplement: Supplementary file 2 — Additional file 2: Figure S1: Stable WI-38 cell lines were subject to a cell viability assay at days 1, 3, 5 and 7 and growth curves were analyzed. Data are shown as mean ± SD. Statistical analysis using one-way ANOVA; ****P ≤0.0001; ***P ≤ 0.001; **P ≤ 0.01. (PDF 99 KB) [file 12885_2014_5171_MOESM2_ESM.pdf]

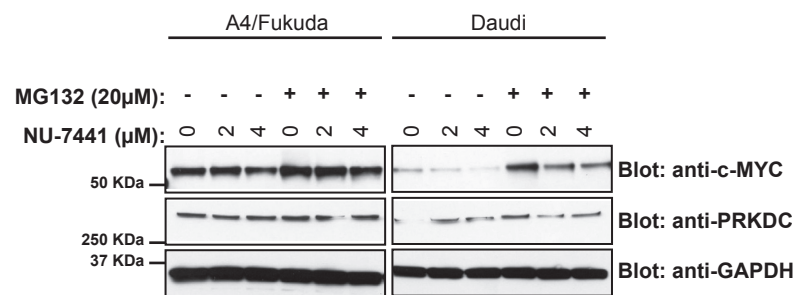

Supplement: Supplementary file 4 — Additional file 4: Figure S3: Different lymphoma cell lines were treated with increasing concentrations of the PRKDC inhibitor, NU-7441, and a proteasome inhibitor, MG132. After a 4 h drug exposure time, c-MYC protein levels were analyzed via immunoblotting with an anti-MYC antibody. PRKDC and GADPH protein levels were also monitored with anti-PRKDC and anti-GADPH antibodies, respectively. (PDF 122 KB) [file 12885_2014_5171_MOESM4_ESM.pdf]
