# Supplementary material for: Identification of synthetic lethality of PRKDC in MYC-dependent human cancers by pooled shRNA screening
Source: BMC Cancer. 2014 Dec 13;14:944. doi: 10.1186/1471-2407-14-944 (PMC4320452; doi:10.1186/1471-2407-14-944)

A

	cell line	NU7441 IC_{50} (µM)	KU0060648 IC_{50} (µM)
high MYC expression	**EB1**	1.71	1.02
	**MC116**	0.64	0.54
	**SU-DHL-10**	0.53	0.95
	**Namalwa**	3.17	1.69
	**Daudi**	1.44	1.04
	**Raji**	2.36	
	**A4/Fukuda**	2.18	0.7
	**WSU-DLCL2**	0.85	0.43
	**OCI-Ly3**	5.9	2.34
low MYC expression	**DEL**	3.49	2.15
	**HUT-102**	8	1.67
	**L428**	3.22	2.7
	**SR786**	5.39	
	**HS604T**	10	7.02
	**HS616T**	37.75	10.29
	**TO175T**	28.47	31.8

B

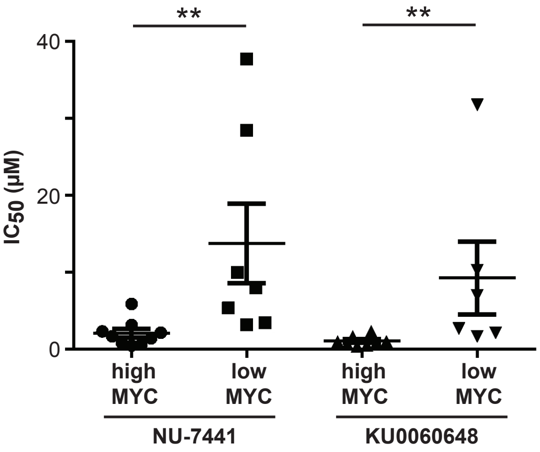

Supplement: Supplementary file 3 — Additional file 3: Figure S2: Human lymphoma cell lines were divided into two groups, high MYC and low MYC gene expression (BioGPS database values), and treated with PRKDC inhibitors, NU-7441 and KU0060648, for 3 days. Cells were subject to cell viability assays and IC50 of the two PRKDC inhibitors were measured in these cell lines. High MYC and low MYC expression cell lines treated with NU-7441 displayed IC50 values of 2.09 + 0.56 μM and 13.76 + 5.18 μM, respectively. The two groups followed a similar trend in terms of IC50 when cells were treated with KU0060648 (1.09 + 0.23 μM and 9.27 + 4.71 μM, respectively). Data are shown as mean ± SEM. Statistical analysis using unpaired t test (Mann-Whitney test); **P ≤ 0.01. (PDF 128 KB) [file 12885_2014_5171_MOESM3_ESM.pdf]
